# Contrast Sensitivity, Quality of Life, and Reading Performance in Glaucoma: A Randomized Crossover Study Comparing Citicoline 500 mg/Homotaurine 50 mg/Nicotinamide 54 mg/Pyrroloquinoline Quinone 5 mg and Citicoline 500 mg

**DOI:** 10.3390/brainsci16101135

**Published:** 2026-10-20

**Authors:** Luca Agnifili, Davide Celani, Matteo Fornaro, Alessandro Sferra, Rodolfo Mastropasqua, Ciro Costagliola

**Affiliations:** 1 Ophthalmology Clinic, Department of Medicine and Ageing Science, University G. d’Annunzio of Chieti-Pescara, 66100 Chieti, Italy; fornaromatteo8@gmail.com (M.F.); alessandro.sferra@studenti.unich.it (A.S.); 2 Alma Mater Studiorum, University of Bologna, 40126 Bologna, Italy; davcel98@gmail.com; 3 Ophthalmology Clinic, Department of Neuroscience, Imaging and Clinical Science, University G. d’Annunzio of Chieti-Pescara, 66100 Chieti, Italy; rodolfo.mastropasqua@unich.it; 4 Department of Neurosciences, Reproductive and Odontostomatological Sciences, University of Naples Federico II, 80138 Naples, Italy; ciro.costagliola@unina.it

**Keywords:** glaucoma, neurodegenerative disease, neuroprotection, citicoline, nicotinamide, pyrroloquinoline quinone, contrast sensitivity, quality of life, reading performance

## Abstract

**Highlights:**

**What are the main findings?**

- CIT/HOMO/B3/PQQ supplementation was associated with favorable short-term changes in contrast sensitivity in early glaucoma.
- Reading performance showed heterogeneous changes, with potentially favorable trajectories for maximum reading speed and reading accessibility.

**What are the implications of the main findings?**

- Contrast sensitivity and reading performance may capture functional changes not detected by conventional glaucoma outcomes.
- Patient-relevant functional measures may complement conventional endpoints in future studies of adjunctive metabolic therapeutic strategies.

**Abstract:**

**Background/Objectives:** This study aimed to compare oral citicoline 500 mg/homotaurine 50 mg/nicotinamide 54 mg/pyrroloquinoline quinone 5 mg (CIT/HOMO/B3/PQQ) with citicoline 500 mg alone (CIT500) for contrast sensitivity (CS), vision-related quality of life (QoL), and reading performance in early, medically controlled primary open-angle glaucoma (POAG). **Methods:** In this prospective, randomized, active-controlled, two-period crossover study, 40 patients (80 eyes) received one supplement for four months before switching to the alternative for four months without washout. Assessments at baseline, month 4 (M4), and month 8 used the Spaeth–Richman Contrast Sensitivity (SPARCS) test, Glaucoma Quality of Life-15 (GQL-15) questionnaire, and digital MNREAD. Bayesian hierarchical linear mixed-effects models evaluated longitudinal changes and between-sequence differences in change from baseline to M4. **Results:** SPARCS increased by 2.52 points (95% credible interval [CrI], 1.04–3.99) at M4 in the CIT/HOMO/B3/PQQ-first sequence and by 3.92 points at month 8 after crossover in the CIT500-first sequence. The randomized M4 contrast favored CIT/HOMO/B3/PQQ (+2.24 points; 95% CrI, 0.16–4.32), whereas corresponding intervals included zero for GQL-15 and all four MNREAD parameters. QoL changes were modest. Maximum reading speed and reading accessibility showed potentially favorable longitudinal trajectories, while reading acuity and critical print size changed less consistently. Intraocular pressure, mean deviation, and retinal nerve fiber layer thickness remained stable. **Conclusions:** CIT/HOMO/B3/PQQ was associated with improved CS and potentially favorable reading-performance trajectories in early POAG. These exploratory findings support further investigation of patient-relevant functional outcomes as complementary measures for evaluating adjunctive therapeutic strategies in glaucoma but require confirmation given the absence of washout and multiplicity adjustment.

## 1. Introduction

Glaucoma is a chronic and progressive neurodegenerative disease characterized by the irreversible loss of retinal ganglion cells (RGCs), leading to visual field (VF) loss and visual disability [1]. Although intraocular pressure (IOP) reduction remains the only proven strategy to manage glaucoma, RGC loss may progress despite adequate IOP control [2].

Even more robust evidence indicates that glaucomatous damage results from multiple interacting risk factors. Mitochondrial dysfunction, oxidative stress, primary vascular dysregulation, and impaired axonal transport have all been implicated in RGC vulnerability, providing a biological rationale for targeting mechanisms beyond IOP reduction [3,4,5].

However, traditional clinical endpoints, such as visual acuity or VF, often fail to capture subtle signs of neurodegenerative processes, particularly in the early stages of disease. In this context, contrast sensitivity (CS) has emerged as a sensitive functional marker, frequently impaired even before detectable changes in conventional functional or structural parameters. The Spaeth–Richman Contrast Sensitivity (SPARCS) test allows evaluation of both central and peripheral CS and has shown a close relationship with visual difficulties encountered in daily life [6,7,8].

Among visual disabilities induced by glaucoma, increasing evidence indicates that measurable reading impairment occurs even in early stages, despite preserved visual acuity. In fact, reduced reading speed, increased critical print size, and higher error rates have been consistently reported using MNREAD charts. In the current digital era, characterized by prolonged engagement in near-vision activities, reading disability may have a substantial impact on daily life, particularly because patients with glaucoma assign high importance to activities requiring central and near vision, including reading [9,10,11,12].

On this basis, CS and reading performance provide complementary measures of visual dysfunction in glaucoma and, together with vision-related quality of life (QoL), may offer a more comprehensive assessment of the functional impact of the disease beyond conventional clinical endpoints [13,14,15]. Previous evidence has shown that specific adjunctive approaches, such as neuroprotective strategies, may contribute to improving CS or QoL in patients with glaucoma. In a multicenter crossover study, supplementation with citicoline 500 mg, homotaurine 50 mg, and vitamin E was associated with improvements in SPARCS and Glaucoma Quality of Life (GQL)-15 scores [16]. Subsequently, a prospective, randomized, crossover study evaluating the fixed combination of citicoline 500 mg and homotaurine 50 mg reported improvements in vision-related QoL assessed by the NEI-VFQ-25 and in inner retinal function, as assessed by pattern electroretinography (PERG) [17].

These results were further confirmed in a newly developed neuroenhancing formulation, that is, the fixed combination of citicoline 500 mg, homotaurine 50 mg, nicotinamide 54 mg, and pyrroloquinoline quinone 5 mg (CIT/HOMO/B3/PQQ), which was designed to target multiple pathogenic mechanisms, including mitochondrial bioenergetics, oxidative stress, and synaptic dysfunction [18]. Preliminary clinical studies have found beneficial effects of this combination on QoL and PERG in patients with stable glaucoma. However, the effects of this formulation on CS and, particularly, on reading performance have not yet been explored. The present prospective, randomized, active-controlled, crossover study was designed to compare the effects of CIT/HOMO/B3/PQQ versus citicoline 500 mg (CIT500) on CS, vision-related QoL, and reading performance in patients with medically controlled glaucoma.

## 2. Materials and Methods

**Figure 1:**
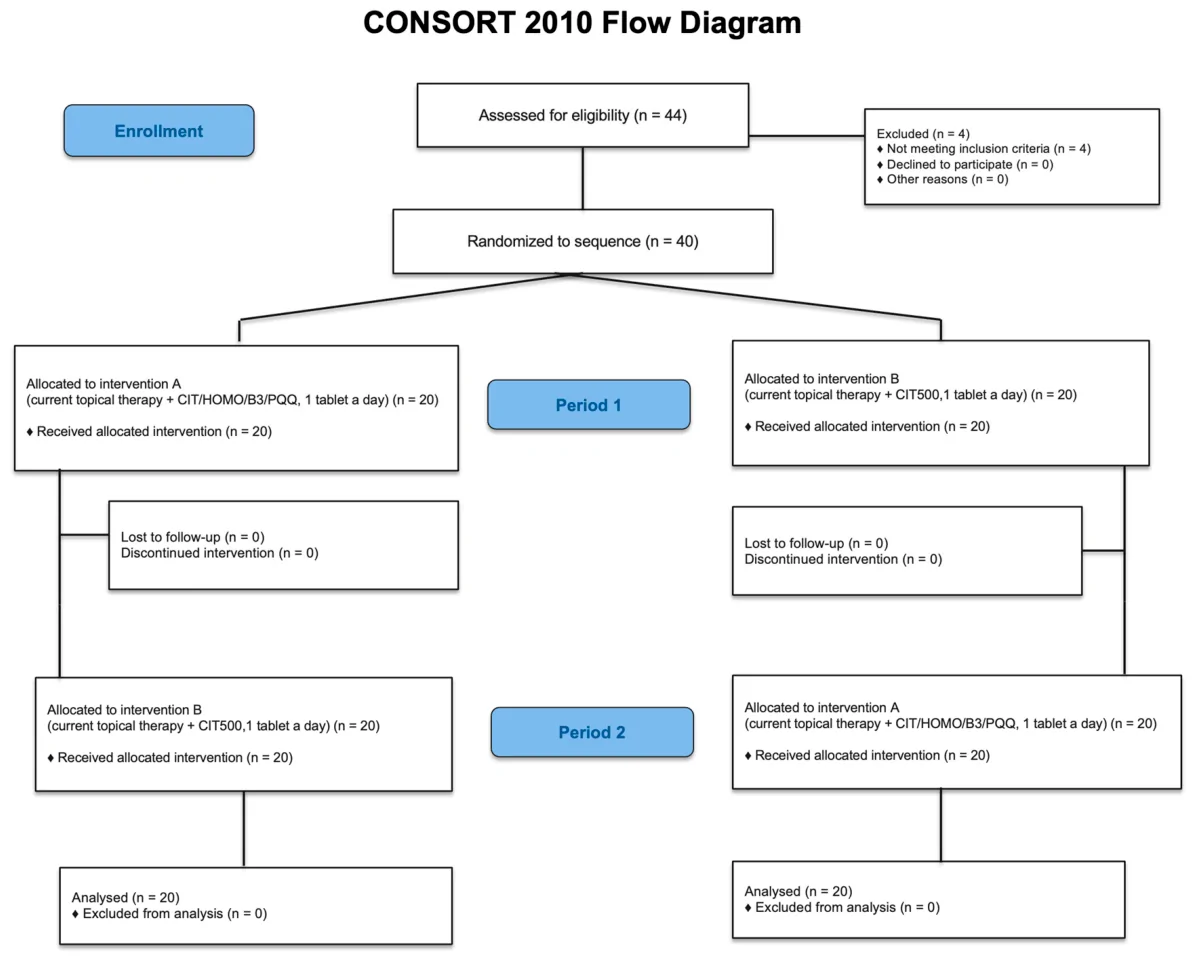
CONSORT 2010 NPFlow.

This was a single-center, prospective, randomized, active-controlled, two-period crossover study, conducted in accordance with the recommendations of the Declaration of Helsinki (revision 2000, Edinburgh, Scotland) and the Italian legislation on good clinical practices (DM 15 July 1997 and amendments). Study approval was obtained from the IRB of the Department of Medicine and Aging Sciences, University G. d’Annunzio of Chieti and Pescara, Chieti, Italy (prot. 36/2024). The study complied with CONSORT 2010 guidelines (Figure 1) and was registered on ClinicalTrials.gov (NCT identifier 07861243). No important changes to the study methods were made after trial commencement. The full study protocol is available from the corresponding author upon reasonable request.

### 2.1. Patients

Adult patients diagnosed with bilateral early primary open-angle glaucoma (POAG) under medical IOP control were consecutively recruited. Glaucoma severity was classified according to the Hodapp–Parrish–Anderson criteria based on VF mean deviation (MD, dB), with early, moderate, and advanced disease defined as MD ≥ −6 dB, MD < −6 to ≥ −12 dB, and MD < −12 dB, respectively [19]. IOP had to be pharmacologically controlled with morning values below 18 mmHg (mean of three measurements with Goldmann applanation tonometry), with any topical therapy (without oral acetazolamide), and stable for at least six months prior to inclusion. Eligible participants were required to have a best-corrected visual acuity of at least 0.5 decimals and a perimetric diagnosis of glaucoma, central corneal thickness ranging from 530 to 570 µm, a visual field (VF) test (24-2 test, full threshold; Humphrey field analyzer II 750; Carl Zeiss Meditec, Inc., Dublin, CA, USA) showing at least three contiguous points on the total deviation probability plot at a level less than 2%, a glaucoma hemifield test ‘‘outside normal limits,’’ and ophthalmoscopic signs of glaucomatous optic disc consistent with the visual field alterations.

Exclusion criteria included moderate to advanced glaucoma, ocular surgery within the previous six months, significant media opacities, retinal or neurological diseases potentially affecting visual performance, systemic disorders influencing visual function, and cognitive impairment interfering with questionnaire administration or reading assessment. Use of other potential neuroprotective agents or nutraceuticals for any indication within the six months preceding enrollment was considered an exclusion criterion. Both eyes of each patient were included in the analysis when they met the eligibility criteria. Patients were randomly assigned in a 1:1 ratio to one of two treatment sequences according to a two-period crossover design. The allocation sequence was generated using a computer-based randomization program. Patients assigned to the CIT/HOMO/B3/PQQ-first sequence received the fixed combination of citicoline 500 mg, homotaurine 50 mg, nicotinamide 54 mg, and PQQ 5 mg (Neuprozin Mito^®^; FB-Vision, San Benedetto del Tronto, AP, Italy) for the first four months, followed by CIT500 (citicoline 500 mg) alone (Optigen Cognizin^®^, Yamamoto Research, Yamamoto S.p.A., Milan, Italy) for an additional four months. Patients assigned to the CIT500-first sequence received the same treatments in the reverse order. Thus, the terms CIT/HOMO/B3/PQQ-first and CIT500-first refer throughout the study to the originally randomized treatment sequence. No washout period was applied between treatment periods. Both supplements were administered orally at a dose of one tablet daily. Evaluations were performed at baseline (M0), month 4 (M4), immediately before treatment crossover, and month 8 (M8), at the end of the second treatment period. Participants were randomly assigned in a 1:1 ratio to one of two treatment sequences using a computer-generated randomization sequence. Simple randomization without blocking or other restrictions was used. The sequence was generated by an investigator not involved in participant enrollment and was concealed from the enrolling investigators until assignment. Participants were enrolled by the study investigators, whereas treatment-sequence assignment was implemented by the enrolling investigator only after eligibility had been confirmed and the concealed allocation had been released. This was an open-label study; therefore, both participants and investigators were aware of the assigned treatment sequence.

### 2.2. Clinical Assessment

At each visit, patients underwent a comprehensive ophthalmological examination, including best-corrected visual acuity measurement, Goldmann applanation tonometry performed between 8:00 and 10:00 a.m., standard automated perimetry, and spectral-domain optical coherence tomography (Cirrus HD-OCT 5000 (Carl Zeiss Meditec, Dublin, CA, USA)) for the assessment of peripapillary retinal nerve fiber layer and ganglion cell layer–inner plexiform layer thicknesses (RNFL-t and GCL-IPL-t). Afterwards, each patient underwent assessment of QoL, CS, and reading performance. At each follow-up visit, safety was assessed by systematically asking patients about any new symptoms or signs, problems related to supplement intake, changes in concomitant medications, and any medical consultations, emergency department visits, or hospitalizations occurring since the previous visit. Treatment adherence was assessed by patient interview and return of the product packages. When a caregiver was present, information regarding regular supplement intake was also obtained from the caregiver. No formal validated adherence instrument or quantitative adherence threshold was used.

### 2.3. Quality of Life Assessment

Vision-related QoL was assessed using the GQL-15 questionnaire, a disease-specific instrument exploring visual difficulties in daily activities. Total scores range from 15 to 75, with higher values indicating worse perceived QoL [14].

### 2.4. Contrast Sensitivity Assessment

CS was assessed binocularly using the SPARCS test on a calibrated computer monitor at a testing distance of 50 cm. The test evaluates central and peripheral CS by presenting vertical square-wave gratings across five visual field regions and provides a total score ranging from 0 to 100, with higher scores indicating better CS. To minimize learning effects, a practice session was performed prior to baseline evaluation [7].

### 2.5. Reading Performance Assessment

Reading performance was evaluated binocularly using the digital MNREAD application (version 1.17, downloaded and installed on an iPad Pro 5th generation) under standardized photopic lighting conditions at a testing distance of 40 cm with appropriate near correction. The test presents standardized sentences in progressively decreasing print sizes. The software automatically calculates reading speed and derives four parameters: reading acuity (RA), defined as the smallest print size read without significant errors; critical print size (CPS), defined as the smallest print sustaining maximum reading speed; maximum reading speed (MRS), representing performance not limited by print size; and the reading accessibility index (RAI), a global measure of functional access to commonly encountered printed material.

### 2.6. Outcome Measures

The primary outcome measures were the changes in total SPARCS score, GQL-15 total score, and the four MNREAD parameters (RA, CPS, MRS, and RAI). Secondary outcome measures included changes in IOP, MD, visual field index (VFI, %), mean peripapillary retinal nerve fiber layer thickness (RNFL-t, µm), and ganglion cell layer–inner plexiform layer thickness (GCL-IPL-t, µm). No changes to the prespecified study outcomes were made after trial commencement.

### 2.7. Statistical Analysis

A Bayesian hierarchical linear mixed-effects model was used to evaluate longitudinal changes across the two randomized treatment sequences. Randomized treatment sequence, visits (M0, M4, and M8), and the sequence-by-visit interaction were included as fixed effects, with M0 serving as the reference visit. The random-effects structure accounted for repeated measurements within subjects and, for eye-level analyses, for correlation between fellow eyes by nesting eye within subject. The sequence-by-visit interaction quantified differences between randomized sequences in change from baseline at each follow-up visit. Because treatment crossover occurred at M4 without a washout period, the M4 interaction contrast represents the randomized between-treatment difference in change during the first treatment period, whereas M8 contrasts were interpreted as sequence-specific longitudinal contrasts rather than isolated treatment effects.

For each primary outcome, the between-sequence difference in change from baseline to M4 was defined as CIT/HOMO/B3/PQQ-first minus CIT500-first and derived from the posterior draws of the fitted models, with results summarized as posterior means and equal-tailed 95% credible intervals. Bayesian analyses were performed using MIRA, an open-source R package for Bayesian analysis of longitudinal and repeated-measures data, with Stan as the underlying Bayesian inference engine [20]. Weakly informative prior distributions were specified for model parameters. Five independent Markov chain Monte Carlo chains were run, each with 2000 warm-up iterations followed by 3000 post-warm-up sampling iterations, yielding 15,000 post-warm-up posterior draws. Sampling and convergence were assessed using CmdStan diagnostic procedures, and all diagnostic checks were satisfactory for the fitted models. Posterior inference was summarized using posterior estimates, 95% credible intervals, and posterior probabilities. No frequentist statistical-significance threshold was applied to the Bayesian analyses.

The sample-size calculation was based on GQL-15 score data reported by Marino et al. [16]. Following the approach described by Senn (Chapter 3) and the formulas reported by Julious (Equations 3.3 and 4.5), the calculation assumed no period or carryover effects and a correlation of approximately r = 0.30 between paired measurements obtained from the same patient [21,22]. Under these assumptions, the standard deviation of the within-patient differences was estimated to be 3.69. A target between-treatment difference of d = 3.4 was specified for sample-size planning, based on an anticipated 10% reduction in the GQL-15 score with CIT/HOMO/B3/PQQ and a 5% reduction with CIT500. For sample-size planning purposes, a frequentist calculation assuming 80% power and a two-sided type I error rate of α = 0.05 yielded a required sample size of 40 patients, with 20 patients allocated to each treatment sequence. The prospective sample-size calculation was based on GQL-15 and was not independently performed for SPARCS or the individual MNREAD parameters. Therefore, adequate power for these outcomes cannot be inferred from the GQL-15-based calculation. The sample-size calculation was performed using the PROC POWER procedure with the TWOSAMPLEMEANS TEST=DIFF option in SAS, version 9.4, TS Level 1M8 (SAS Institute Inc., Cary, NC, USA).

The outcomes were analyzed using separate outcome-specific Bayesian models, and no formal multiplicity adjustment was applied across GQL-15, SPARCS, and the four MNREAD parameters. Accordingly, posterior estimates and 95% credible intervals represent marginal, outcome-specific summaries rather than simultaneous multiplicity-adjusted inference across endpoints.

## 3. Results

### 3.1. Study Population

Forty patients (80 eyes) with early and IOP-controlled POAG were enrolled and randomized to the two treatment sequences (20 patients each). Participants were recruited between November 2024 and June 2025, and follow-up was completed in February 2026. No adverse events or treatment discontinuations were reported during the study period, and all patients completed the 8-month follow-up. The study was completed as planned and was not stopped prematurely.

The overall mean age was 65.6 ± 13.36 years (66.7 ± 12.12 and 64.6 ± 12.0 years in the CIT/HOMO/B3/PQQ-first and CIT500-first groups, respectively), and 23 patients (57.5%) were male. Demographic and clinical characteristics were comparable between the two treatment-sequence groups at baseline (Table 1 and Table 2).

### 3.2. Clinical Parameters

IOP, MD, and RNFL-t did not change during follow-up in either group. GCL-IPL-t showed a small decrease at M4 in the CIT/HOMO/B3/PQQ-first sequence (−1.09 µm; 95% CrI, −2.16 to −0.01), whereas at M8 the 95% CrI included zero (−0.97 µm; 95% CrI, −2.04 to 0.11). VFI did not change in the CIT/HOMO/B3/PQQ-first sequence, whereas in the CIT500-first sequence it increased by 2.50% at M4 (95% CrI, 0.88 to 4.13) and remained 1.64% above baseline at M8 (95% CrI, 0.03 to 3.28) (Table 1).

### 3.3. Contrast Sensitivity

**Figure 2:**
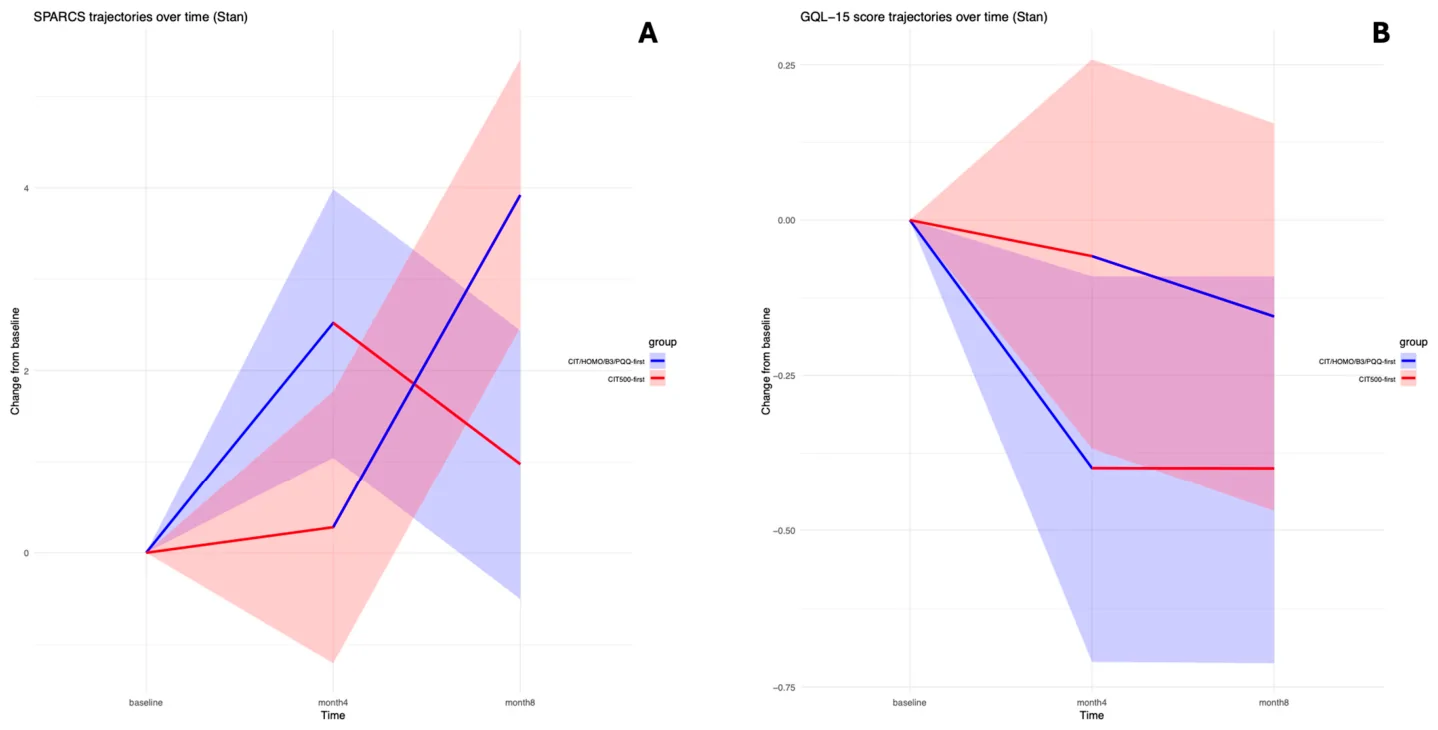
**Changes in contrast sensitivity and vision-related quality of life during the two treatment sequences.** Longitudinal changes in Spaeth–Richman Contrast Sensitivity (SPARCS) (**A**) score and Glaucoma Quality of Life-15 (GQL-15) (**B**) score from baseline (M0) to month 4 (M4) and month 8 (M8) in the CIT/HOMO/B3/PQQ-first and CIT500-first sequences. At M4, treatments were crossed over (from M4 to M8, each group received the alternative treatment). Group labels indicate the initially randomized treatment sequence. Higher SPARCS scores indicate better contrast sensitivity, whereas lower GQL-15 scores indicate better vision-related QoL.

At M4, SPARCS score increased by 2.52 points (95% CrI, 1.04–3.99) in the CIT/HOMO/B3/PQQ-first sequence, indicating improved CS compared with baseline, whereas only a small increase (+0.28 points) was observed in the CIT500-first sequence. After treatment crossover, the two groups showed opposite trajectories: in patients switching from CIT/HOMO/B3/PQQ to CIT500, the improvement observed at M4 was attenuated at M8 (+0.97 points versus baseline), whereas patients switching from CIT500 to CIT/HOMO/B3/PQQ showed a further increase in SPARCS score, reaching +3.92 points versus baseline at M8 (Table 2, Figure 2).

### 3.4. Vision-Related QoL

The GQL-15 score decreased by 0.40 points at M4 in the CIT/HOMO/B3/PQQ-first sequence, indicating an improvement in vision-related QoL, whereas only a minimal change (−0.06 points) was observed in the CIT500-first sequence. Following crossover, the improvement observed in the CIT/HOMO/B3/PQQ-first group was substantially maintained, while the CIT500-first group showed a further reduction in GQL-15 score after switching to CIT/HOMO/B3/PQQ (Table 2, Figure 2).

### 3.5. Reading Performance

At M4, RA remained substantially unchanged in the CIT/HOMO/B3/PQQ-first sequence (+0.01 logMAR), whereas it decreased by 0.05 logMAR in the CIT500-first sequence. At M8, RA showed only a minimal change from baseline in the CIT/HOMO/B3/PQQ-first sequence (−0.01 logMAR), whereas the reduction observed at M4 in the CIT500-first sequence was maintained and slightly increased (−0.07 logMAR).

CPS decreased by 0.06 logMAR at M4 in the CIT/HOMO/B3/PQQ-first sequence, while it remained essentially unchanged (+0.01 logMAR) in the CIT500-first sequence. At M8, CPS was 0.03 logMAR above baseline in the CIT/HOMO/B3/PQQ-first sequence and 0.08 logMAR below baseline in the CIT500-first sequence.

MRS increased from baseline at M4 in both treatment sequences by 7.30 words/min (95% CrI, 2.47–12.14) in the CIT/HOMO/B3/PQQ-first group and by 8.06 words/min (95% CrI, 3.47–12.65) in the CIT500-first group. After crossover, the trajectories diverged: in the CIT/HOMO/B3/PQQ-first sequence, the increase in MRS was attenuated to +2.18 words/min versus baseline at M8, whereas in the CIT500-first sequence, MRS further increased to +11.64 words/min versus baseline.

**Figure 3:**
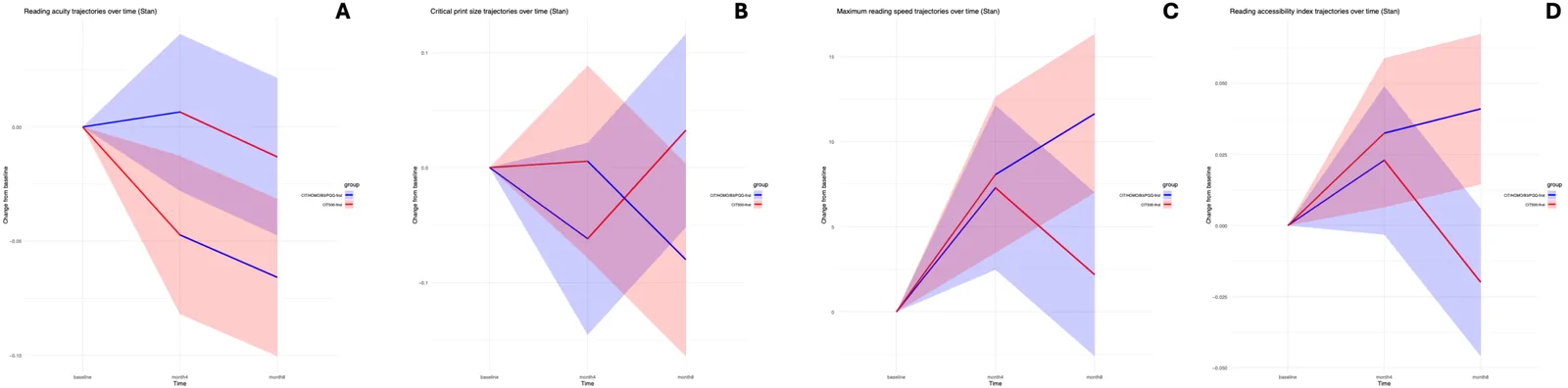
**Changes in reading performance during the two treatment sequences.** Longitudinal changes in reading acuity (RA) (**A**), critical print size (CPS) (**B**), maximum reading speed (MRS) (**C**), and reading accessibility index (RAI) (**D**), assessed using MNREAD, from baseline (M0) to month 4 (M4) and month 8 (M8) in the CIT/HOMO/B3/PQQ-first and CIT500-first sequences. At M4, treatments were crossed over; from M4 to M8, each group received the alternative treatment. Group labels indicate the initially randomized treatment sequence. Lower RA and CPS values indicate better performance, whereas higher MRS and RAI values indicate better performance.

RAI increased at M4 in both treatment sequences, by 0.02 in the CIT/HOMO/B3/PQQ-first group and by 0.03 in the CIT500-first group. At M8, RAI was 0.02 below baseline in the CIT/HOMO/B3/PQQ-first sequence, whereas in the CIT500-first sequence, it further increased to +0.04 versus baseline (Table 2, Figure 3).

The between-sequence difference in mean change from baseline to M4 favored the CIT/HOMO/B3/PQQ-first sequence for SPARCS (+2.24 points; 95% CrI, 0.16–4.32), whereas the 95% credible intervals included zero for GQL-15 and all four MNREAD parameters (Table 3).

## 4. Discussion

The present prospective crossover study investigated the effects of oral CIT/HOMO/B3/PQQ supplementation compared with CIT500 on CS, vision-related QoL, and reading performance in patients with early, medically controlled POAG. The main finding was a favorable functional effect of CIT/HOMO/B3/PQQ on CS, together with more heterogeneous changes in QoL and reading performance.

In particular, MRS and RAI showed potentially favorable trajectories during exposure to CIT/HOMO/B3/PQQ, whereas RA and CPS behaved less consistently. These findings extend previous observations with citicoline/homotaurine-based combinations by introducing reading performance as a novel functional outcome [16,17,18].

Previous clinical studies of citicoline/homotaurine-based combinations have mainly focused on electrophysiological retinal function. Cornelio et al. reported increased PERG amplitudes during supplementation with citicoline/homotaurine/vitamin E, which were reversed after treatment withdrawal, while conventional clinical parameters remained stable [23]. Similarly, Rossi et al. documented improvements in PERG parameters with citicoline/homotaurine [17]. More recently, the same CIT/HOMO/B3/PQQ formulation evaluated in the present study showed more favorable effects than citicoline 800 mg alone on selected P50 and N95 parameters, together with higher NEI-VFQ25 total and selected domain scores [18]. Collectively, these studies suggest that functional changes may become detectable over relatively short periods and provide a rationale for investigating whether multi-component formulations may exert effects beyond those associated with citicoline alone.

Conversely, functional and structural parameters, including MD, VFI, RNFL-t, and GCL-IPL-t, remained substantially stable throughout the study. The small fluctuations observed in VFI and GCL-IPL-t should be interpreted cautiously, as short-term test–retest and measurement variability cannot be distinguished from true structural or functional change, particularly given the short follow-up and the early stage of glaucoma in our population.

Our CS findings are particularly consistent with the QUALICOS study, in which citicoline/homotaurine/vitamin E increased SPARCS scores by approximately 8–10% and reduced GQL-15 scores by approximately 13–14% in patients with mild POAG [16]. In the present study, SPARCS similarly represented the clearest functional signal: improvement during CIT/HOMO/B3/PQQ exposure was attenuated after switching to CIT500 and became more pronounced when patients initially receiving CIT500 crossed over to CIT/HOMO/B3/PQQ. Although this pattern should be interpreted cautiously, it is directionally consistent with previous findings and supports CS as a potentially sensitive functional endpoint for short-term studies of complementary neuroprotective or neuromodulatory strategies in glaucoma [16].

The biological rationale for CIT/HOMO/B3/PQQ is particularly relevant to mitochondrial and metabolic dysfunction, increasingly recognized as a key contributor to RGC vulnerability in glaucoma [3,4,5,24]. RGCs are highly energy-dependent neurons, and impaired mitochondrial bioenergetics, oxidative phosphorylation, and redox homeostasis may reduce their ability to withstand glaucomatous stress [25]. Citicoline has pleiotropic neuroprotective and neuromodulatory actions, including effects on neuronal membrane integrity, neurotransmission, oxidative stress, and mitochondrial function [26]. Of particular interest, nicotinamide is a precursor of NAD+, a critical cofactor for cellular energy metabolism whose depletion may increase RGC susceptibility, while nicotinamide supplementation has shown robust protection in experimental glaucoma and preliminary functional benefits in patients [24,25,27]. PQQ may provide complementary mitochondrial support by promoting NADH oxidation to NAD+, NAD+-dependent SIRT1/PGC-1α signaling, mitochondrial biogenesis, adenosine triphosphate (ATP) production, and antioxidant pathways [4]. Thus, the combination provides a biological rationale for simultaneously targeting neuronal function, redox homeostasis, and mitochondrial resilience. Nevertheless, the present findings represent evidence of functional modulation rather than direct demonstration of neuroprotection, since neither the underlying cellular mechanism nor an effect on long-term RGC survival can be established from our data.

The principal novelty of the present study is the evaluation of reading performance, a clinically relevant and potentially under-recognized manifestation of glaucoma that may be impaired despite relatively preserved visual acuity [9,28]. Reading requires integration of contrast perception, central and paracentral visual information, fixation and eye movements, visual span, and higher-order visual processing [12,29]. In an increasingly digitalized environment, these functions are particularly relevant because reading and near-vision activities represent a substantial component of everyday visual demands.

MRS and RAI provided the most interesting reading-related findings. MRS increased by 7.30 wpm during the first four months of CIT/HOMO/B3/PQQ, but the gain was attenuated after switching to CIT500. Conversely, MRS increased by 8.06 wpm during initial CIT500 treatment and reached +11.64 wpm after crossover to CIT/HOMO/B3/PQQ. RAI showed a broadly similar sequence-dependent trajectory, with loss of the initial improvement after withdrawal of CIT/HOMO/B3/PQQ and persistence or further improvement when the combination was introduced. Although these findings do not establish treatment superiority or identify the contribution of individual components, they are compatible with an additional functional effect of CIT/HOMO/B3/PQQ beyond citicoline alone. The mitochondrial-active components nicotinamide and PQQ may provide complementary metabolic support through NAD+-dependent bioenergetics, mitochondrial homeostasis, and ATP availability, potentially enhancing RGC functional efficiency under conditions of high metabolic demand [4,24,25]. This interpretation remains hypothesis-generating.

The different behavior of RA and CPS may reflect the distinct dimensions captured by MNREAD: these parameters predominantly reflect print-size-related thresholds, whereas MRS and RAI more directly characterize suprathreshold reading efficiency and functional access to text [12,29]. Accordingly, subtle changes in visual processing may be more readily expressed by speed- and accessibility-related measures. The concomitant favorable changes in SPARCS and these reading parameters are also intriguing, since reduced CS, VF loss, restricted visual span, crowding, and oculomotor abnormalities contribute to glaucoma-related reading disability [12,29]. Improved contrast perception or visual processing efficiency might therefore facilitate fluent reading without necessarily modifying threshold reading acuity. However, this possible functional relationship remains hypothesis-generating because correlations between changes in SPARCS and MNREAD parameters were not specifically investigated.

The clinical magnitude of these functional changes should, however, be interpreted cautiously. SPARCS has demonstrated high test–retest reliability (ICC = 0.97), although Richman et al. reported a coefficient of repeatability of ±6.7% [7]. Thus, the mean SPARCS changes observed in the present study (+2.52 points at M4 in the CIT/HOMO/B3/PQQ-first sequence and +3.92 points at M8 in the CIT500-first sequence) are smaller than the published repeatability threshold, although a practice session was performed before baseline assessment to minimize learning effects. Similarly, published MNREAD repeatability data in adults reported a coefficient of repeatability of approximately 47 words/min for maximum reading speed, substantially larger than the mean MRS changes observed in our study (+7.30 to +11.64 words/min) [30]. However, these published repeatability estimates were obtained in populations and testing settings different from those of the present study and therefore cannot be directly applied as thresholds to patients with early POAG assessed using digital MNREAD. Moreover, established minimal clinically important differences for SPARCS and MNREAD outcomes specifically in glaucoma are lacking. Accordingly, our findings should be interpreted as evidence of favorable group-level functional trajectories rather than as demonstrating clinically meaningful improvement at the individual-patient level.

Changes in GQL-15 were less consistent than those observed for SPARCS, although the early improvement during CIT/HOMO/B3/PQQ supplementation is consistent with previous GQL-15 and NEI-VFQ25 findings with citicoline/homotaurine-based combinations [16,17,18]. Patient-reported outcomes integrate multiple dimensions of visual disability and may be less sensitive than performance-based tests to relatively small functional changes over short periods [13,14,15]. Moreover, the early and stable glaucoma in our population may have limited the magnitude of measurable improvement in perceived disability.

Several limitations should be acknowledged. The sample size, although prospectively calculated, was relatively limited, and the short follow-up and restriction to patients with bilateral early and relatively symmetric POAG limit the generalizability of our findings and do not allow conclusions regarding long-term disease modification. This is particularly relevant to the interpretation of reading performance, which was assessed binocularly. Therefore, whether similar functional effects would be observed in patients with unilateral, markedly asymmetric, moderate, or advanced glaucoma, in whom binocular visual function may be differently affected, remains to be determined. In addition, no formal adjustment for multiplicity was applied across the multiple functional outcomes. Therefore, although the original designation of the outcomes remains unchanged, favorable findings for individual endpoints should be interpreted as exploratory evidence requiring confirmation rather than as multiplicity-controlled confirmatory evidence of efficacy. The prospective sample-size calculation was based on GQL-15 and was not independently performed for SPARCS or the individual MNREAD parameters; accordingly, adequate power for these outcomes cannot be inferred from the GQL-15-based calculation.

The absence of a placebo or untreated control does not completely exclude repeated-testing, learning, or expectation effects. The use of CIT500 as an active comparator was chosen because the primary aim of the study was not to assess supplementation versus no treatment, but rather to explore whether the multicomponent formulation provided additional functional effects beyond those associated with citicoline alone. This design allowed the contribution of the additional components to be investigated against a common citicoline background; however, it does not permit determination of the absolute treatment effect of either intervention relative to placebo or no supplementation. The absence of a washout period also raises the possibility of residual or carryover effects after crossover, particularly for outcomes that remained improved after the treatment switch. Accordingly, M8 findings should be interpreted as sequence-specific longitudinal changes following sequential exposure to both interventions rather than as isolated effects of the treatment administered during the second period. Greater inferential weight should therefore be placed on the randomized between-treatment comparison at M4. In addition, repeated MNREAD testing may have introduced a learning effect. Finally, the study cannot determine which component of CIT/HOMO/B3/PQQ accounts for any effect beyond that associated with citicoline.

Taken together, CIT/HOMO/B3/PQQ supplementation was associated with a favorable effect on CS and potentially beneficial changes in selected components of reading performance in patients with early, medically controlled POAG. The randomized M4 comparison favored CIT/HOMO/B3/PQQ for SPARCS but provided no clear evidence of between-treatment differences for GQL-15 or MNREAD outcomes, and these findings remain exploratory given the absence of multiplicity adjustment. Importantly, the behavior of MRS and RAI extends previous evidence by suggesting that reading efficiency may represent a sensitive and clinically meaningful functional outcome in glaucoma.

## 5. Conclusions

The present findings provide evidence of short-term functional modulation associated with CIT/HOMO/B3/PQQ supplementation and encourage continued investigation of mitochondrial dysfunction as a therapeutic target in glaucoma, particularly through metabolic approaches involving nicotinamide and PQQ. Reading performance emerges as a potentially useful patient-relevant functional endpoint that may complement conventional measures in future studies of adjunctive therapeutic strategies. However, the present study does not demonstrate neuroprotection or disease modification, which would require longer follow-up and evidence of sustained structural or functional preservation. Further larger and longer-term studies are needed to confirm these findings, investigate their underlying mechanisms, and determine whether similar functional effects can be observed in patients with more advanced glaucoma.

## Figures and Tables

**Table 1:** Baseline and follow-up clinical features in treatment groups.

	Baseline	4 Months	8 Months
IOP (mmHg)			
*CIT/HOMO/B3/PQQ-FIRST*	14 (12/17.50)	0.12 (95% CrI: −0.63, 0.86)	−0.73 (95% CrI: −1.47, 0.01)
*CIT500-FIRST*	15 (14.5/16)	−0.36 (95% CrI: −1.11, 0.37)	0.31 (95% CrI: −0.43, 1.05)
MD (dB)			
*CIT/HOMO/B3/PQQ-FIRST*	−2.54 (−5.275/−1.685)	−0.29 (95% CrI: −0.93, 0.35)	−0.30 (95% CrI: −0.94, 0.34)
*CIT500-FIRST*	−2.56 (−6.00/−1.08)	0.39 (95% CrI: −0.24, 1.01)	0.23 (95% CrI: −0.39, 0.86)
VFI (%)			
*CIT/HOMO/B3/PQQ-FIRST*	95 (85/97.5)	−0.33 (95% CrI: −1.97, 1.30)	0.66 (95% CrI: −0.97, 2.30)
*CIT500-FIRST*	93 (88/98)	2.50 (95% CrI: 0.88, 4.13) ^‡^	1.64 (95% CrI: 0.03, 3.28) ^‡^
RNFL-t (µm)			
*CIT/HOMO/B3/PQQ-FIRST*	70.50 (64/76.75)	−1.10 (95% CrI: −2.53, 0.32)	−0.89 (95% CrI: −2.31, 0.54)
*CIT500-FIRST*	76.50 (68.25/85.00)	−0.62 (95% CrI: −2.03, 0.81)	−0.32 (95% CrI: −1.74, 1.09)
GCL-IPL-t (µm)			
*CIT/HOMO/B3/PQQ-FIRST*	68 (60.5/73.5)	−1.09 (95% CrI: −2.16, −0.01)	−0.97 (95% CrI: −2.04, 0.11)
*CIT500-FIRST*	67 (64.5/73)	−0.73 (95% CrI: −1.81, 0.34)	−0.20 (95% CrI: −1.28, 0.87)

IOP: intraocular pressure; MD: mean deviation; VFI: visual field index; RNFL-t: retinal nerve fiber layer thickness; GCL-IPL-t: ganglion cell layer–inner plexiform layer thickness. Treatment groups refer to the randomized treatment sequence. At month 4, treatments were crossed over: patients in the CIT/HOMO/B3/PQQ-first group switched to CIT500, whereas those in the CIT500-first group switched to CIT/HOMO/B3/PQQ; ^‡^ indicates posterior evidence of improvement from baseline, defined according to the clinically favorable direction of change for each outcome. Accordingly, improvement may correspond to either a decrease or an increase in the outcome value. Posterior evidence was considered present when the 95% credible interval for the change from baseline lay entirely on the clinically favorable side of zero. Baseline values are expressed as median and interquartile range (Q1–Q3); follow-up values represent estimated changes from baseline with 95% credible intervals.

**Table 2:** Baseline and follow-up contrast sensitivity, quality of life, and reading performance in treatment groups.

	Baseline	4 Months	8 Months
SPARCS			
*CIT/HOMO/B3/PQQ-FIRST*	76 (71\78.5)	+2.52 (95% CrI: 1.04–3.99) ^‡^	+0.97 (95% CrI: −0.50–2.44)
*CIT500-FIRST*	77 (75\81)	+0.28 (95% CrI: −1.20–1.77)	+3.92 (95% CrI: 2.47–5.41) ^‡^
GQL−15			
*CIT/HOMO/B3/PQQ-FIRST*	19.50 (15\22.25)	−0.40 (95% CrI: −0.71 to −0.09) ^‡^	−0.40 (95% CrI: −0.71 to −0.09) ^‡^
*CIT500-FIRST*	18.50 (17.50\22.25)	−0.06 (95% CrI: −0.37 to 0.26)	−0.16 (95% CrI: −0.47 to 0.16)
Reading Acuity			
*CIT/HOMO/B3/PQQ-FIRST*	0 (−0.10\0.20)	0.01 (95% CrI: −0.03 to 0.04)	−0.01 (95% CrI: −0.05 to 0.02)
*CIT500-FIRST*	0 (−0.10\0.15)	−0.05 (95% CrI: −0.08 to −0.01) ^‡^	−0.07 (95% CrI: −0.10 to −0.03) ^‡^
Critical Print Size			
*CIT/HOMO/B3/PQQ-FIRST*	0.30 (0.15\0.70)	−0.06 (95% CrI: −0.15, 0.02)	0.03 (95% CrI: −0.05, 0.12)
*CIT500-FIRST*	0.30 (0.20\0.40)	0.01 (95% CrI: −0.08, 0.09)	−0.08 (95% CrI: −0.16, 0.00)
Maximum Reading Speed			
*CIT/HOMO/B3/PQQ-FIRST*	138 (131\143)	7.30 (95% CrI: 2.47–12.14) ^‡^	2.18 (95% CrI:−2.60–7.01)
*CIT500-FIRST*	130 (115\143.5)	8.06 (95% CrI: 3.47–12.65) ^‡^	11.64 (95% CrI: 7.00–16.33) ^‡^
Reading Accessibility Index			
*CIT/HOMO/B3/PQQ-FIRST*	0.67 (0.61\0.70)	0.02 (95% CrI: 0.00, 0.05) ^‡^	−0.02 (95% CrI: −0.05, 0.01)
*CIT500-FIRST*	0.64 (0.57\0.68)	0.03 (95% CrI: 0.01, 0.06) ^‡^	0.04 (95% CrI: 0.01, 0.07) ^‡^

SPARCS: Spaeth–Richman Contrast Sensitivity; GQL-15: Glaucoma Quality of Life-15. Treatment groups refer to the randomized treatment sequence. At month 4, treatments were crossed over: patients in the CIT/HOMO/B3/PQQ-first group switched to CIT500, whereas those in the CIT500-first group switched to CIT/HOMO/B3/PQQ; ^‡^ indicates posterior evidence of improvement from baseline, defined according to the clinically favorable direction of change for each outcome. Accordingly, improvement may correspond to either a decrease or an increase in the outcome value. Posterior evidence was considered present when the 95% credible interval for the change from baseline lay entirely on the clinically favorable side of zero. Baseline values are expressed as median and interquartile range (Q1–Q3); follow-up values represent estimated changes from baseline with 95% credible intervals.

**Table 3:** Between-sequence differences in change from baseline to month 4 for the primary outcomes.

Outcome	Unit	Posterior Mean CIT/HOMO/B3/PQQ–CIT500	95% Crl Lower Limit	95% Crl Upper Limit
SPARCS score	Points	2.24	0.16	4.32
GQL-15 score	Points	−0.34	−0.78	0.10
Reading Acuity (RA)	logMAR	0.05	0.00	0.10
Critical Print Size (CPS)	logMAR	−0.07	−0.18	0.05
Maximum Reading Speed (MRS)	Words/min	−0.77	−7.11	5.60
Reading Accessibility Index (RAI)	dimensionless	−0.01	−0.05	0.03

Contrast was defined as the change in the CIT/HOMO/B3/PQQ-first sequence minus the change in the CIT500-first sequence and derived from the posterior draws of the Bayesian models fitted using MIRA; values are posterior means with equal-tailed 95% credible intervals. SPARCS: Spaeth–Richman Contrast Sensitivity; GQL-15: Glaucoma Quality of Life-15.

## Data Availability

The data supporting the findings of this study are available from the corresponding author upon reasonable request.

## Abbreviations

The following abbreviations are used in this manuscript:

ATP	Adenosine triphosphate
CIT500	Citicoline 500 mg
CIT/HOMO/B3/PQQ	Citicoline 500 mg/homotaurine 50 mg/nicotinamide 54 mg/pyrroloquinoline quinone 5 mg
CPS	Critical print size
CrI	Credible interval
CS	Contrast sensitivity
GCL-IPL-t	Ganglion cell layer–inner plexiform layer thickness
GQL-15	Glaucoma Quality of Life-15
IOP	Intraocular pressure
IRB	Institutional Review Board
MD	Mean deviation
MNREAD	MNREAD reading acuity test
MRS	Maximum reading speed
NAD+	Nicotinamide adenine dinucleotide, oxidized form
NADH	Nicotinamide adenine dinucleotide, reduced form
NEI-VFQ-25	National Eye Institute Visual Function Questionnaire-25
PERG	Pattern electroretinogram
POAG	Primary open-angle glaucoma
PQQ	Pyrroloquinoline quinone
QoL	Quality of life
RA	Reading acuity
RAI	Reading accessibility index
RGC	Retinal ganglion cell
RNFL-t	Retinal nerve fiber layer thickness
SPARCS	Spaeth–Richman Contrast Sensitivity
VF	Visual field
VFI	Visual Field Index
wpm	Words per minute

## References

[1] Weinreb R.N., Aung T., Medeiros F.A. (2014). The pathophysiology and treatment of glaucoma: A review. JAMA.

[2] Heijl A., Leske M.C., Bengtsson B., Hyman L., Bengtsson B., Hussein M. (2002). Early Manifest Glaucoma Trial Group. Reduction of intraocular pressure and glaucoma progression: Results from the Early Manifest Glaucoma Trial. Arch. Ophthalmol..

[3] Mastropasqua L., Agnifili L., Ferrante C., Sacchi M., Figus M., Rossi G.C.M., Brescia L., Aloia R., Orlando G. (2022). Citicoline/coenzyme Q10/vitamin B3 fixed combination exerts synergistic protective effects on neuronal cells exposed to oxidative stress. Nutrients.

[4] Medoro A., Davinelli S., Mazzotta C.G., Agnifili L., Scapagnini G. (2026). Pyrroloquinoline quinone and NAD+ metabolism in glaucoma: A molecular rationale for combined neuroprotection. Pharmaceuticals.

[5] Tezel G. (2021). Multifactorial pathogenic processes of retinal ganglion cell degeneration in glaucoma towards multi-target strategies for broader treatment effects. Cells.

[6] Lahav K., Levkovitch-Verbin H., Belkin M., Glovinsky Y., Polat U. (2011). Reduced mesopic and photopic foveal contrast sensitivity in glaucoma. Arch. Ophthalmol..

[7] Richman J., Zangalli C., Lu L., Wizov S.S., Spaeth E., Spaeth G.L. (2015). The Spaeth/Richman contrast sensitivity test (SPARCS): Design, reproducibility and ability to identify patients with glaucoma. Br. J. Ophthalmol..

[8] Sun Y., Erdem E., Lyu A., Zangalli C., Wizov S.S., Lo D., Spaeth E.E., Richman J., Spaeth G.L. (2016). The SPARCS: A novel assessment of contrast sensitivity and its reliability in patients with corrected refractive error. Br. J. Ophthalmol..

[9] Ishii M., Seki M., Harigai R., Abe H., Fukuchi T. (2013). Reading performance in patients with glaucoma evaluated using the MNREAD charts. Jpn. J. Ophthalmol..

[10] Rolle T., Dallorto L., Cafasso R., Mazzocca R., Curto D., Nuzzi R. (2019). Reading ability in primary open-angle glaucoma: Evaluation with Radner Reading Charts. Optom. Vis. Sci..

[11] Ikeda M.C., Hamada K.U., Bando A.H., Nakamura V.P.L., Prata T.S., Tatham A.J., Paranhos A., Gracitelli C.P.B. (2021). Interventions to improve reading performance in glaucoma. Ophthalmol. Glaucoma.

[12] Goddin T.L., Yu H., Friedman D.S., Owsley C., Kwon M. (2023). MNREAD reading vision in adults with glaucoma under mesopic and photopic conditions. Investig. Ophthalmol. Vis. Sci..

[13] Floriani I., Quaranta L., Rulli E., Katsanos A., Varano L., Frezzotti P., Rossi G.C.M., Carmassi L., Rolle T., Ratiglia R. (2016). Health-related quality of life in patients with primary open-angle glaucoma: An Italian multicentre observational study. Acta Ophthalmol..

[14] Nelson P., Aspinall P., Papasouliotis O., Worton B., O’Brien C. (2003). Quality of life in glaucoma and its relationship with visual function. J. Glaucoma.

[15] Freeman E.E., Muñoz B., West S.K., Jampel H.D., Friedman D.S. (2008). Glaucoma and quality of life: The Salisbury Eye Evaluation. Ophthalmology.

[16] Marino P.F., Rossi G.C.M., Campagna G., Capobianco D., Costagliola C. (2020). Effects of citicoline, homotaurine, and vitamin E on contrast sensitivity and visual-related quality of life in patients with primary open-angle glaucoma: A preliminary study. Molecules.

[17] Rossi G.C.M., Rolle T., De Silvestri A., Sisto D., Mavilio A., Mirabile A.V., Paviglianiti A., Strano B., Picasso E., Pasinetti G.M. (2022). Multicenter, prospective, randomized, single blind, cross-over study on the effect of a fixed combination of citicoline 500 mg plus homotaurine 50 mg on pattern electroretinogram (PERG) in patients with open angle glaucoma on well controlled intraocular pressure. Front. Med..

[18] Rossi G.C.M., Rinaldi M., Matarazzo F., Strianese D., Campagna G., La Ragione M., Esposito Veneruso P., Scapagnini G., Costagliola C. (2025). Randomized, cross over, multicenter, single-blind study comparing citicoline 500 mg/homotaurine 50 mg/vitamin B3 54 mg/pyrroloquinoline quinone 5 mg (Neuprozin Mito^®^) and citicoline 800 mg (Cebrolux^®^) on pattern electroretinogram (PERG) and quality of life in patients with primary open-angle glaucoma with well-controlled intraocular pressure. J. Clin. Med..

[19] Hodapp E., Parrish R.K., Anderson D.R. (1993). Clinical Decisions in Glaucoma.

[20] Celani D. (2026). MIRA: Bayesian Multilevel Inference for Longitudinal Data, Version v0.0.1.

[21] Senn S. (1993). Cross-Over Trials in Clinical Research.

[22] Julious S.A. (2010). Sample Sizes for Clinical Trials.

[23] Cornelio C., Crisigiovanni L., Limardo V., Nuzzo D., Troiano P. (2021). Neuroprotection in primary open-angle glaucoma: The role of a fixed citicoline-homotaurine-vitamin E combination. Int. J. Ophthalmol. Vis. Sci..

[24] Williams P.A., Harder J.M., Foxworth N.E., Cochran K.E., Philip V.M., Porciatti V., Smithies O., John S.W.M. (2017). Vitamin B3 modulates mitochondrial vulnerability and prevents glaucoma in aged mice. Science.

[25] Tribble J.R., Otmani A., Sun S., Ellis S.A., Cimaglia G., Vohra R., Jöe M., Lardner E., Venkataraman A.P., Domínguez-Vicent A. (2021). Nicotinamide provides neuroprotection in glaucoma by protecting against mitochondrial and metabolic dysfunction. Redox Biol..

[26] Faiq M.A., Wollstein G., Schuman J.S., Chan K.C. (2019). Cholinergic nervous system and glaucoma: From basic science to clinical applications. Prog. Retin. Eye Res..

[27] Ju W.K., Perkins G.A., Kim K.Y., Bastola T., Choi W.Y., Choi S.H. (2023). Glaucomatous optic neuropathy: Mitochondrial dynamics, dysfunction and protection in retinal ganglion cells. Prog. Retin. Eye Res..

[28] Ikeda M.C., Bando A.H., Hamada K.U., Nakamura V.P.L., Prata T.S., Paranhos A., Tatham A.J., Gracitelli C.P.B. (2021). Is reading performance impaired in glaucoma patients with preserved central vision?. J. Glaucoma.

[29] Kwon M., Liu R., Patel B.N., Girkin C. (2017). Slow reading in glaucoma: Is it due to the shrinking visual span in central vision?. Investig. Ophthalmol. Vis. Sci..

[30] Mataftsi A., Bourtoulamaiou A., Haidich A.B., Antoniadis A., Kilintzis V., Tsinopoulos I.T., Dimitrakos S. (2013). Development and validation of the Greek version of the MNREAD acuity chart. Clin. Exp. Optom..

